# Supplementary material for: Endoscopic Ultrasound-Guided Radiofrequency Ablation as an Future Alternative to Pancreatectomy for Pancreatic Metastases from Renal Cell Carcinoma: A Prospective Study
Source: Cancers (Basel). 2021 Oct 20;13(21):5267. doi: 10.3390/cancers13215267 (PMC8582413; doi:10.3390/cancers13215267)
Supplement: Supplementary file 1 [file cancers-13-05267-s001.zip › cancers-1394185-supplementary.pdf]

Supp Figure S1 - Study detail

Supp Figure S2 - Time until progression for all treated PM. Sub-group analysis according to PM size

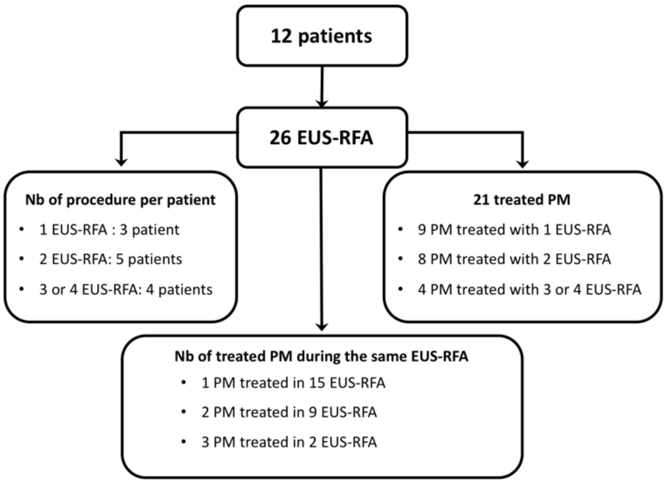

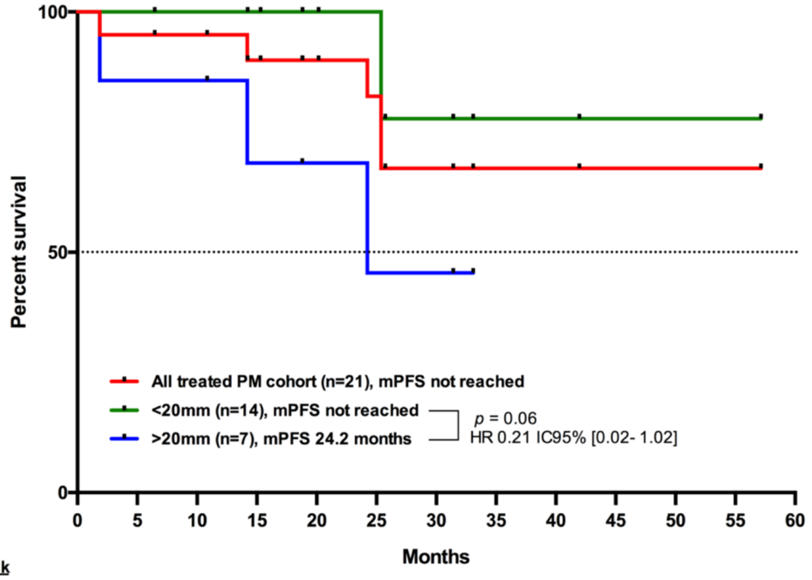

<u>Patients at Risk</u>

All PM Cohort	21	20	20	19	14	13	10	5	5	3	3	3	0
PM size < 20mm	14	14	14	13	11	10	7	4	4	2	2	2	0
PM size > 20mm	7	6	6	5	4	3	3	1	1	1	1	1	0

Supp Table S1- EUS-RFA procedures characteristics

n = 26			
Median number of procedures per patient	n (range)	2	[1-4]
Median time between procedures (months)	n (range)	3.6	[2.5-15.2]
Median Number of Procedures per PM	All PM n (range)	2	[1-4]
	> 20mm n (range)	1	[1-4]
	≤ 20mm n (range)	1	[1-2]
Median number of PM treated in each EUS-RFA	n (range)	1	[1-3]
Post **EUS-RFA** severe Side Effects	Immediate (n, %)	0	0
	Delayed (n, %)	2	8

SuppTable S2: Focal control at 2, 6 and 12 months according to PM size

	< 20mm (%) (n=14)			> 20mm (%) (n=7)		
	**2mo.**	**6mo.**	**12mo.**	**2mo.**	**6mo.**	**12mo.**
**FOCAL CONTROL RATE**	92,9	91.7	72.7	85.7	71.4	75
**COMPLETE RESPONSE**	28,6	33.3	45.5	43.9	14.3	25
**PROGRESSION**	7.1	8.3	27.3	14.3	28.6	25
**NOT EVALUABLE**	0.0	14.3	21.4	0.0	0.0	42.9

PM: pancreatic metastasis CR: complete response, PR: partial response, SD: stable disease PD: progression disease, mo.: Months

Focal Control Rate = CR + PR +SD

Supp Table S3: Logistic regression of variables potentially associated with 6-months objective response.

	6 months Response	
	OR (95%IC)	P-value
**Age**<br>*(for 5 years)*	1.44 (0.39-6.17)	0.57
**Sex**<br>*(male vs female)*	0.50 (0.07-3.13)	0.46
**Tumor Localization**<br>*(tail vs body)*<br>*(head-uncus vs body)*	 0.50 (0.03-6.95)<br>0.62 (0.06-5.22)	 0.60<br>0.66
**Tumor size**<br>*(≥20mm vs <20mm)*	0.37 (0.04-2.50)	0.31
**IMDC**<br>*(Intermediate vs good)*	0.11 (0.009-0.92)	0.057

IMDC= International Metastatic RCC Database Consortium; OR = Odds Ratio.
